# Effect of Vitamin D_2_ Fortification Using *Pleurotus ostreatus* in a Whole-Grain Cereal Product on Child Acceptability

**DOI:** 10.3390/nu11102441

**Published:** 2019-10-14

**Authors:** Cristina Proserpio, Vera Lavelli, Francesca Gallotti, Monica Laureati, Ella Pagliarini

**Affiliations:** Department of Food, Environmental and Nutritional Sciences (DeFENS), University of Milan, 20133 Milan, Italy; vera.lavelli@unimi.it (V.L.); francesca.gallotti@unimi.it (F.G.); monica.laureati@unimi.it (M.L.); ella.pagliarini@unimi.it (E.P.)

**Keywords:** liking, mushroom, food neophobia, vitamin D, sustainability, acceptability

## Abstract

Vitamin D_2_ deficiency is one of the most common micronutrient insufficiencies among children. Few foods, mainly those derived from animal sources, naturally contain this vitamin. The basidiomycete mushroom *Pleurotus ostreatus* could be used as an innovative and sustainable ingredient for food fortification with vitamin D_2_. This study was aimed at exploring children’s acceptance of a whole-cereal-based product (breadsticks) combined with increasing concentrations of *P. ostreatus* powder rich in vitamin D_2_. The food neophobia trait (fear of trying unfamiliar and new food) on sample acceptability was also investigated. One hundred and three children (47 girls and 56 boys, aged 9–11 years) were recruited, and breadstick-liking was studied in relation to gender and neophobic traits. Results showed that the samples enriched in vitamin D_2_ were well accepted by children even if liking decreased with increasing concentration of mushroom powder. Generally, neophilic subjects gave higher liking scores compared with the neophobic ones, especially for the modified samples. New, well-accepted fortified products could be developed using an adequate concentration of mushroom powder to deal with the increasing vitamin D_2_ deficiency among children.

## 1. Introduction

Vitamin D deficiency is recognized to be a worldwide problem [1], which has raised the need to develop new dietary strategies to increase its intake. Vitamin D has a well-known role related to calcium absorption and homeostasis, bone mineralization, and bone health. Additionally, a nuclear receptor for the bioactive form of vitamin D (i.e., 1,25(OH)_2_D) is present in at least 38 human tissues and organs [2] and is probably related to the risk-reduction potential of vitamin D towards other diseases. These diseases include psoriasis, multiple sclerosis, inflammatory bowel disease, type 1 and 2 diabetes, hypertension, cardiovascular disease, metabolic syndrome, and various cancers [3].

Based on these considerations, it is crucial to maintain, both by sunlight exposure and by dietary intake [4], an adequate vitamin D status. Indeed, sunlight exposure that leads to the endogenous production of this vitamin is not sufficient to reach the recommended vitamin D daily intake. This is especially true in specific populations, such as African Americans with dark skin pigmentation which decreases UVB exposure, as well as elderly people with a low level of vitamin D_3_ precursor [1]. In both adults and children more than one year old, the recommended vitamin D intake is 15 μg/day [5]. Since few foods are a source of this vitamin, food enrichment is considered a useful strategy to achieve this recommendation. In most studies, the prevalent form of vitamin D used for food enrichment was vitamin D_3_, which is found only in animal sources such as fish oil and liver. However, vitamin D_2_, which mainly comes from mushrooms, can provide a more sustainable alternative, considering the low environmental impact of mushroom production. Among mushrooms belonging to basidiomycete phyla, the *Pleurotus* genus is recognized as having an interesting nutritional profile due to its valuable essential amino acid scoring pattern and high β-glucan content, as well as important micronutrients concentrations. This mushroom presents a high level of some vitamins of the B group and vitamin D_2_—the latter up to 5.93 µg/g in the *Pleurotus ostreatus* species [6]. Thus, *P. ostreatus* appears to be an interesting natural source of vitamin D_2_ for specific consumer groups, including vegetarians, vegans, and people intolerant to lactose, since most of the fortified products with vitamin D_2_ available on the market are dairy-based products. Moreover, *P. ostreatus* is able to grow efficiently on low-cost substrates such as lignocellulosic agri-food byproducts, leading to the possible production of sustainable food ingredients [7,8,9].

However, mushroom powder addition to foods elicits sensory attributes that could potentially affect product acceptability [7]. It has been highlighted that liking of vegetable soups where *P. ostreatus* powder was added was affected in adult subjects. In particular, consumers’ liking decreased as the concentration of mushroom powder increased [8]. Other findings revealed that unfortified wheat pasta obtained the lowest liking scores, while acceptability increased with the addition of a low amount of the insoluble β-glucan fraction from *P. ostreatus* powder [10]. It has recently been shown that a cereal-based product (flatbread) enriched with *P. ostreatus* powder obtained higher liking scores compared with the control sample without mushroom addition in a group of adolescents [9]. Thus, it is clear that developing new food formulations is challenging considering that food perception and food-liking—one of the main drivers of food consumption—differs greatly among individuals. Moreover, developing food products with optimized nutritional and sensory characteristics is of crucial importance, especially for specific target populations such as children, who show well-known neophobic reactions [11]. Food neophobia, defined as the reluctance to eat unfamiliar foods [12], is considered a negative eating behavior because it can decrease diet variety and reduce daily fruit and vegetable intake [13]. This behavior also seems to be related to the nutritional status of subjects, considering neophobic individuals may be less willing to try healthy alternative versions of familiar products, leading to weight gain [14].

The aim of the present study was to investigate children’s acceptance of a product based on whole-wheat cereal supplemented with increasing concentrations of *P. ostreatus* powder rich in vitamin D_2_. The influence of food neophobic traits on sample acceptability was also investigated.

Breadsticks were chosen as the enriched food prototype, since they could be easily consumed as snacks by children during the day, and because cereal-based products are generally well accepted by this specific target population [15,16]. Moreover, since breadsticks were made with whole-wheat flour, they could also be used to meet the recommended intake of whole-grain foods, considering the reported low consumption in children [17].

## 2. Materials and Methods

### 2.1. Participants

One hundred and three children (47 girls and 56 boys, aged 9–11 years) were recruited in the Milan area (Italy) via primary schools. Parents received full information about the research study and provided written informed consent for their children’s participation. Children without a signed informed consent were not involved in the study. Children who suffered from food allergies or did not like the tested samples, which could negatively affect hedonic responses, performed alternative activities so that they did not feel excluded from the test activities. The study was performed in adherence with the principles established by the Declaration of Helsinki. The protocol was approved by the Ethics Committee of the University of Milan (protocol n°19/18).

### 2.2. Stimuli

A commercial strain of *P. ostreatus* deposited at the mycotheca of the Società Agricola IoBoscoVivo (Vergiate, Varese, Italy) was used. This strain was cultivated on wood of *Populus tremula* in a greenhouse at room temperature. After one year of incubation, about 10 kg of body fruits were collected, cut into 4-mm slices, spread on an oven rack in a single layer, and air-dried at 37 °C for 48 h. At the end of the process, about 1 kg of dried mushroom was obtained and all the product was ground to a fine powder using a Thermomix TM 31 (Vorwerk Contempora S.r.l., Italy). The production of breadsticks added with *P. ostreatus* was performed at the Società Agricola IoBoscoVivo (Vergiate, Varese, Italy), according to the addition levels decided on by the sensory group at the University of Milan, based on preliminary trials. The breadstick ingredients were whole-wheat flour, sunflower oil, yeast, and salt. The experimental samples were prepared by adding different increasing concentrations of *P. ostreatus* powder (B_2_ = 2%, B_4_ = 4%, and B_6_ = 6%) to the standard formulation (B_0_). A hidden control of the unmodified sample (B_hc_ = 0%) was also included to verify that children were able to assign a comparable score to the two samples without mushroom powder addition (B_0_ and B_hc_). The nutritional composition and the energy content of the developed samples, which were provided by Società Agricola IoBoscoVivo (Vergiate, Varese, Italy), are reported in Table 1.

Approximately 30 g of each sample were presented at room temperature to the children in plastic bags labelled with three-digit codes. Water was available for rinsing the palate.

### 2.3. Determination of Vitamin D_2_ Content

Vitamin D_2_ was extracted according to the procedure of Sławinska and collaborators [18] and identified by the HPLC procedure by Huang and colleagues [6]. Analysis was performed on breadsticks enriched with 2%, 4%, and 6% of *P. ostreatus* powder and on the control breadstick after processing and on the breadstick enriched with 6% of *P. ostreatus* after storage at room temperature for six months.

### 2.4. Experimental Procedure

The liking evaluation was performed at school in the classroom in the presence of a teacher and an experimenter in a single day. During the evaluation, each child was seated at their own table and received the questionnaire, which was extensively explained by the experimenters. The questionnaire consisted of an evaluation of food neophobia followed by the evaluation of breadstick liking. The questionnaire was self-completed by the children. To increase ecological validity, the breadsticks were offered as a snack and liking was assessed during the mid-morning break. Each of the five formulations, coded with three-digit numbers, were randomly presented to each child in blind condition. Children were instructed to drink a sip of water between each sample. The experimenters monitored the children to ensure that they did not influence each other during the evaluation. The session took approximately 30 min and children received a small award (e.g., pencil case) for their participation.

### 2.5. Food Neophobia Assessment

The Italian Children Food Neophobia Scale (ICFNS), previously validated by Laureati and collaborators [11] with a large sample of school-aged children, was used to investigate children’s neophobic traits. The ICFNS consists of eight items, and it is a simplification of the original Food Neophobia Scale (FNS) of Pliner and Hobden [12] composed of 10 items. Each item offers five graded responses by a facial expression, in order to help the child better understand the level of agreement or disagreement for each item alternatives, from “very false for me” (score 1) to “very true for me” (score 5). Half of the items represent neophobic whereas the other half represent neophilic food situations. Thus, responses to neophilic statements were reversed when calculating the score. The ICFNS score was calculated as a sum of the responses, yielding a range of 8–40.

### 2.6. Liking Assessment

Children were asked to taste the products and to express their liking scores using a seven-point facial hedonic scale from “super good” (score 7) to “super bad” (score 1) [19] which has been proven to be a reliable tool to be used with young consumers [20]. The experimenters provided instructions for the use of the scale prior to tasting and during the session.

### 2.7. Data Analysis

Data on vitamin D_2_ content were analyzed by one-way ANOVA using the least significant difference (LSD) as a multiple range test. Results are reported as average ± standard error (SE).

A preliminary linear mixed model procedure was carried out on overall liking data considering samples (B_0_, B_2_, B_4_, B_6_, and B_hc_), gender (female and male), and their interactions as fixed factors. Age was added to the model as a covariate. Considering that this first analysis showed that the liking scores of sample B_0_ and the control (B_hc_) were not statistically different, B_hc_ was not considered in the subsequent analysis. Another model was constructed following the previous approach on food neophobia data.

To determine the influence of food neophobia on liking scores, a linear mixed model was carried out with liking data as the dependent factor, and food neophobia levels (low, medium, high), sample, gender, and their interactions as fixed factors. Subjects were considered as random factors in all the analyses. When a significant difference (*p* < 0.05) was found, the LSD post hoc test was performed as a multiple comparison test. All statistical analyses were performed using IBM SPSS Statistics for Windows, Version 24.0 (IBM Corp., Armonk, NY, USA).

## 3. Results

### 3.1. Vitamin D_2_ Content and Product Design

The vitamin D_2_ contents of the developed breadsticks are reported in Table 2.

As shown in Table 2, the addition of *P. ostreatus* from 2% to 6% significantly increased the level of vitamin D_2_ from not detectable to 28.4 µg in 100 g. Breadsticks enriched with 2%, 4%, and 6% can provide 32%, 54%, and 95% of the recommended daily amount of vitamin D_2_ per single dose (50 g). The 6% enriched breadsticks were also analyzed after being stored for six months at room temperature and found to have the same vitamin D_2_ content as just after production. According to the USDA database, milk and orange juice fortified with vitamin D provide 30% of the Recommended Dietary Allowance (RDA), while 20% and 10% of RDA is provided by fortified yogurt and cereal breakfast, respectively (https://ods.od.nih.gov/factsheets/VitaminD-HealthProfessional/#h3). Hence, all the enriched breadsticks could be of interest to improve the dietary intake of vitamin D.

### 3.2. Liking Assessment

**Figure 1:**
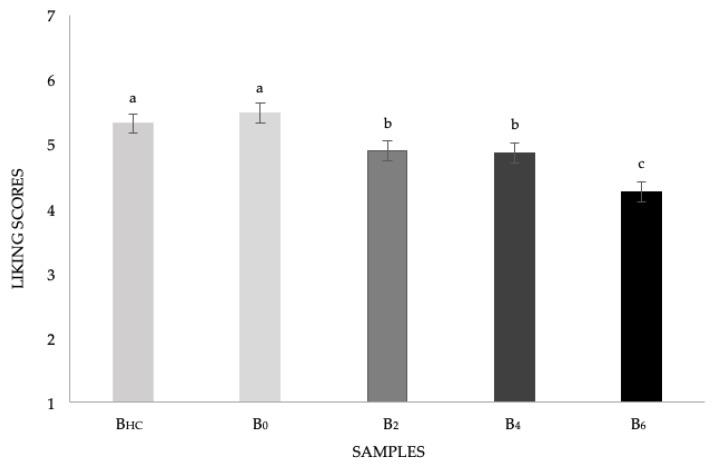
Mean liking scores ± SEM by samples. Different letters indicate significant differences according to post hoc test. B_hc_ and B_0_ = 0%; B_2_ = 2%; B_4_ = 4%; and B_6_ = 6% of mushroom powder.

A significant sample effect was found on liking scores (F_(4; 404)_ = 12.38, *p* < 0.0001). As reported in Figure 1, B_0_ and B_hc_ samples obtained comparable scores (M_B0_ = 5.5 ± 0.2; M_Bhc_ = 5.3 ± 0.2). Children equally liked these samples, which were significantly preferred to samples with increasing added concentrations of *P. ostreatus* powder. Samples enriched with mushroom powder at 2% and 4% obtained comparable liking scores to each other (M_B2_ = 4.9 ± 0.2; M_B4_ = 4.9 ± 0.1) while the sample with the highest concentration of *P. ostreatus* powder received the lowest liking scores (M_B6_ = 4.3 ± 0.2). However, all the vitamin D_2_-enriched samples were well accepted by the children (liking scores were still up the middle of the scale).

The main factor “gender” and the interaction “sample*gender” were not significant (F_(1;141)_ = 0.01, *p* = 0.9; F_(4;410)_ = 0.9, *p* = 0.5, respectively).

Satisfactory internal consistency, as calculated through Cronbach’s alpha test (alpha = 0.75), was observed among the ICFNS items. The mean food neophobia value of the children was 19.5 ± 0.3. No significant differences in neophobic traits were found between girls and boys (F_(1;101)_ = 0.42, *p* = 0.52).

To investigate the relationship between food neophobic traits and breadstick formulation liking, children were categorized according to their neophobia scores into the following three groups, after verifying that data were normally distributed [11]: children with scores in the lower 25th percentile of ICFNS scores, score < 16, *n* = 23 (LOW_FN); children with scores between the 25th and 75th percentiles, 16 ≤ ICFNS score ≤ 23, *n* = 59 (MEDIUM_FN); and children with scores in the upper 25th percentile, ICFNS score > 23, *n* = 21 (HIGH_FN).

**Figure 2:**
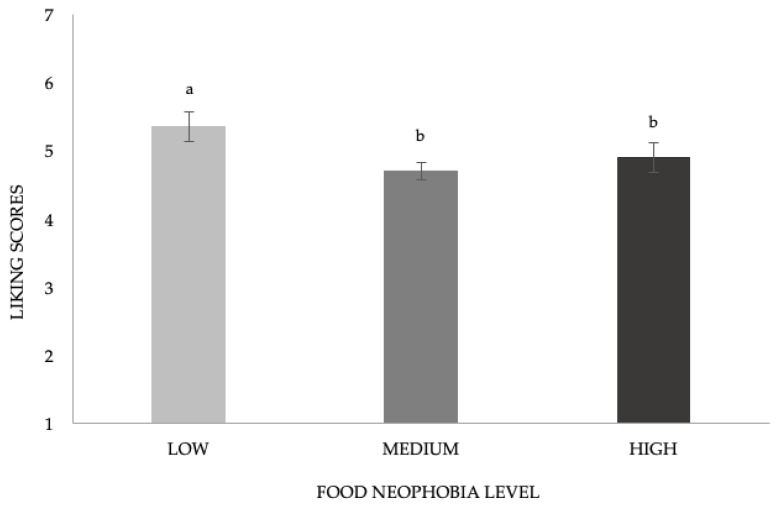
Mean liking scores ± SEM according to food neophobia levels. Different letters indicate significant differences according to post hoc test.

A significant effect of food neophobia on liking scores was found (F_(2;98)_ = 3.32, *p* = 0.04). As reported in Figure 2, children categorized by LOW_FN gave generally significantly higher liking scores (M = 5.3 ± 0.2) compared with MEDIUM_FN (M = 4.7 ± 0.1) and HIGH_FN (M = 4.9 ± 0.2).

**Figure 3:**
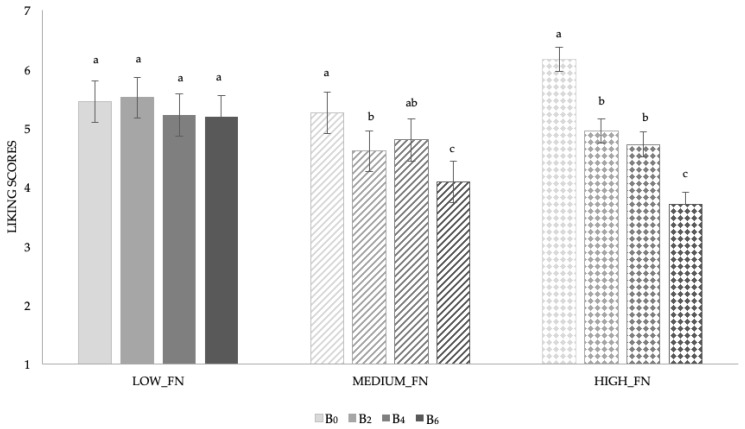
Mean liking scores ± SEM by samples according to food neophobia levels. Different letters indicate significant differences according to post hoc test for each sample. B_0_ = 0%; B_2_ = 2%; B_4_ = 4%; and B_6_ = 6% of mushroom powder.

In Figure 3 the results of the interaction “sample*food neophobia levels”, which was also significant (F_(6;291)_ = 2.33, *p* = 0.03), are reported.

Looking at liking scores obtained by the children categorized according to their food neophobia level, no differences were found in sample liking scores in the LOW_FN group. Interestingly, results obtained by the HIGH_FN children showed that they gave significantly lower liking scores to sample B_2_ (M = 4.9 ± 0.3) and B_4_ (M = 4.7 ± 0.4), which were comparable to each other, than the unmodified sample (M_B0_ = 6.2 ± 0.2). Neophobic children gave the lowest liking rating (M = 3.7 ± 0.3) to the sample with the highest concentration of mushroom sample. A similar trend was highlighted for the MEDIUM_FN children.

## 4. Discussion

The recommended intake of vitamin D has been defined as 15 µg/day [5]. There is no general consensus as to whether the mushroom source D_2_ is equally as effective as the animal source D_3_. Indeed, some studies have demonstrated that vitamin D_3_ is more efficient than vitamin D_2_ [21], while other studies have shown that vitamin D_2_ and vitamin D_3_ treatment yield equivalent outcomes in the treatment of hypovitaminosis D among young children [22]. What can be stated is that plant sources of vitamin D_2_ are more sustainable than D_3_ sources of animal origin [23].

Aside from the different sources of vitamin D_2_ and D_3_, issues regarding compliance with the dietary regimen have also been highlighted. For instance, in a study by Johnson and colleagues [24], cheese was fortified with 17.6 μg of vitamin D_3_/100 g showing an effect in increasing vitamin D status. However, there were five dropouts due to gastrointestinal problems, a dislike for the saltiness of the cheese, and medical advice due to the relatively high levels of salt and saturated fat provided by the portion of fortified cheese (85 g) to be ingested. In a study by Daly and collaborators [25], milk fortified by 5.0 μg of vitamin D_3_/100 g caused some dropouts due to the large volume of milk (400 mL/day) required to be consumed.

In the present study, whole-grain breadsticks were used as a model food to be enriched with vitamin D_2_ from *P. ostreatus*. This represents a sustainable vitamin source because it can grow efficiently on various clean by-products of food processing and has low production costs. Therefore, this mushroom represents a value-added ingredient for food fortification. The breadsticks developed in this study could also be conveniently sized in the right amount and provide a significant percentage of the recommended daily level of both vitamin D and dietary fiber, since these products could be easily consumed as snacks during the day.

The liking results of the present study demonstrated that even if the acceptability decreased with increasing concentration of mushroom powder, all the samples were clearly well accepted by the children. Thus, the developed food could be useful to integrate the vitamin D_2_ daily intake, which depends on clinical and environmental factors such as latitude of residence, level of exposure to the sun, and dietary practices [26]. In this context, consuming fortified food could be a complementary strategy to sunlight exposure and the ingestion of some food that naturally contains vitamin D_2_ to meet recommended vitamin daily intake. Different types of fortified food have already been developed, such as fortified milk, cheese, soy drink, and fish [27]. However, these mentioned products might not be suitable for subjects who are intolerant to lactose or for who consume low amounts of fish, such as children [28]. The low fish consumption reported among children could be associated with behavioral factors such as food neophobia, which shape the development of fish disliking [29]. Thus, it is important to develop new foods enriched in vitamin D_2_ considering children’s food preferences, habits, food intolerance, and neophobic traits.

The present results demonstrate that children characterized by a high food neophobia score were less prone to accept the enriched samples, especially with regard to the breadsticks with the highest amount of mushroom powder. Although low liking ratings were highlighted for this product, acceptability could be increased by enhancing its familiarity through repeated exposure. Indeed, it is widely recognized that repeated exposure to a specific food increases the liking and consumption of that food due to a “learned safety” behavior [30,31]. Thus, the repeated consumption of an unfamiliar food without undesirable consequences leads to increased acceptance of that food. Neophilic children, and partially children with medium levels of neophobia, showed acceptance of all the developed breadstick samples, even the one with the higher amount of mushroom powder.

Some limitations of the study should be mentioned. The present results provide evidence of product acceptability, but no suggestions about which sensory characteristics are related to hedonic perceptions. This information could be useful during product development in order to optimize the sensory quality. Thus, a future perspective of study could be to consider a sensory description of fortified model foods enriched with *P. ostreatus* powder in order to identify drivers of liking and disliking of these products. Moreover, it could be interesting to add the mushroom powder in other food products and investigate consumers’ responses in other specific target populations, such as elderly people. Indeed, it has been widely reported that elderly subjects show a direct relation between vitamin D levels and frailty syndrome as well as an inverse association between vitamin D levels and the risk of oral, gastrointestinal, urinary, ocular, and respiratory infections [32]. Finally, even if samples were provided to the children in blind condition and in a randomized order, there could be other factors such as social determinants as well as the children’s natural preference toward the snack (breadsticks) that also might influence their responses.

In conclusion, the findings of the present study suggest that it is feasible to develop new food formulations using an adequate concentration of mushroom powder rich in vitamin D_2_ that could also satisfy children’s preferences. The developed fortified foods could be considered more suitable to prevent vitamin D_2_ deficiency in children than the fortified products already available on the market. Indeed, the developed breadsticks could potentially be launch on the market as a healthy snack since they received a positive hedonic response by the young consumers involved.

## Figures and Tables

**Table 1:** Nutritional values (g per 100 g dry weight) and energy content (kcal per 100 g) of the developed breadsticks.

Nutritional Values	Samples			
	B_0_	B_2_	B_4_	B_6_
Energy	390 kcal	384 kcal	372 kcal	372 kcal
Fat	11.7 g	11.7 g	11.6 g	11.6 g
Saturated fat	1.3 g	1.3 g	1.2 g	1.25 g
Carbohydrates	64.6 g	64.5 g	64.2 g	64.2 g
Proteins	12.2 g	12.6 g	13.3 g	13.3 g
Fiber	11 g	11 g	11.5 g	11.5 g
Salt	0.4 g	0.4 g	0.4 g	0.4 g

**Table 2:** Vitamin D_2_ content of breadsticks enriched with *Pleurotus ostreatus*.

Samples	µg in 100 g	µg per Dose (50 g)	% Daily Dose
B_0_	N.d.		
B_2_	9.5 ^a^ ± 0.3	4.8	32
B_4_	16.3 ^b^ ± 0.2	8.2	54
B_6_	28.4 ^c^ ± 1.6	14.2	95
B_6_, six months stored	27.1 ^c^ ± 0.9	14.2	95

N.d. = not detectable. Values with different superscripts in column are significantly different (LSD, *p* < 0.05).
